# Comparing the analgesic effect of heat patch containing iron chip and ibuprofen for primary dysmenorrhea: a randomized controlled trial

**DOI:** 10.1186/1472-6874-12-25

**Published:** 2012-08-22

**Authors:** Shahindokht Navvabi Rigi, Fatihe kermansaravi, Ali Navidian, Leila Safabakhsh, Ameneh Safarzadeh, Somaye Khazaian, Shahla Shafie, Tahmineh Salehian

**Affiliations:** 1 Pregnancy Health Center, Zahedan University of Medical Sciences, Zahedan, Iran

## Abstract

**Background:**

Primary dysmenorrhea is a common and sometimes disabling condition. In recent years, some studies aimed to improve the treatment of dysmenorrhea, and therefore, introduced several therapeutic measures. This study was designed to compare the analgesic effect of iron chip containing heat wrap with ibuprofen for the treatment of primary dysmenorrhea.

**Methods:**

In this randomized (IRCT201107187038N2) controlled trial, 147 students (18–30 years old) with the diagnosis of primary dysmenorrhea were enrolled considering the CONSORT guideline. Screening for primary dysmenorrhea was done by a two-question screening tool. The participants were randomly assigned into one of the intervention groups (heat Patch and ibuprofen). Data regarding the severity and emotional impact of the pain were recorded by a shortened version of McGill Pain Questionnaire (SF-MPQ). Student's *t* test was used for statistical analysis.

**Results:**

The maximum and minimum pain severities were observed at 2 and 24 hours in both groups. The severity of sensual pain at 8, 12, and 24 hours was non-significantly less in the heat Patch group. There was also no significant difference between the groups regarding the emotional impact of pain at the first 2, 4, 8, 12 and 12 hours of menstruation.

**Conclusions:**

Heat patch containing Iron chip has comparable analgesic effects to ibuprofen and can possibly be used for primary dysmenorrhea.

**Trial registration:**

IRCT201107187038N2

## Background

Primary dysmenorrhea is a common and sometimes disabling condition among women of childbearing age [1] The prevalence of dysmenorrhea worldwide is, with rates ranging from 15.8-89.5%, with higher prevalence rates reported in adolescent populations [2] The prevalence is estimated to be 72% among menstruating women in Iran [3]. Dysmenorrhea is caused by excessive uterine contraction, interruption of blood supply, and production of prostaglandins. During the first 24 hours of menstruation, endometrial vessels contract, and blood supply to the endometrial tissues is reduced, which eventually leads to necrosis of the endometrial layer [4]. Pain and lower abdominal cramps are among the most common causes of gynaecological referrals [5-7]. Dysmenorrhea is sometimes associated with nausea, vomiting, diarrhea, fatigue, fever, headache [6,8,9], back pain, and dizziness [1].

Factors contributing to dysmenorrhea include the age of early menarche, increased menstrual bleeding, alcohol and tobacco use, low socioeconomic status, obesity, and depression [10]. Childbirth decreases the likelihood of dysmenorrhea because of the reduced number of adrenergic receptors in the uterus [4-11]. Dysmenorrhea is the leading cause of absence from school and work and has a negative impact on the quality of life and general health of women [8,12-14]. It is also a major burden to the family's economic status [6].

In recent years, some researchers have aimed to improve the treatment of dysmenorrhea. Several therapeutic measures have been introduced, including the use of non-steroidal anti-inflammatory drugs (NSAIDs) as first-line therapy [15], and some other measures such as local heat [1,7]. The analgesic effect of local heat is similar to the electrical cutaneous stimulation of nerves, which is explained by the gate-control theory of pain or by central alteration of the pain threshold [16,17].

Thermal therapy has traditionally been used to treat dysmenorrhea [16,17]. Usual forms of thermal therapy such as hot water bottles and electric pads may be annoying and may interfere with daily life. Although oral medications are effective, they can cause constipation and other gastrointestinal symptoms and should be administered only if justified by the severity of pain [17]. For instance, ibuprofen can cause gastrointestinal inflammation or bleeding, skin rash, pruritus, tinnitus, dizziness, and renal or hepatic complications [18].

Even though pharmacological treatment of dysmenorrhea is usually successful, the failure rate is about 20–25% [6]. NSAIDs are the first choice of treatment, but sometimes side effects such as adverse gastrointestinal symptoms oblige patients to look for alternative treatments. Although heat (in different forms such as a hot bag, towel, or bottle) has traditionally been used to ease pain in many cultures, its utilization is currently limited because of the lack of interest among youth in traditional remedies and because of the limited research undertaken to evaluate its effectiveness.

We aimed to compare a non-pharmaceutical, non-invasive treatment (heat wrap) with a routine medication (ibuprofen) for the treatment of treat dysmenorrhea.

## Methods

**Figure 1:**
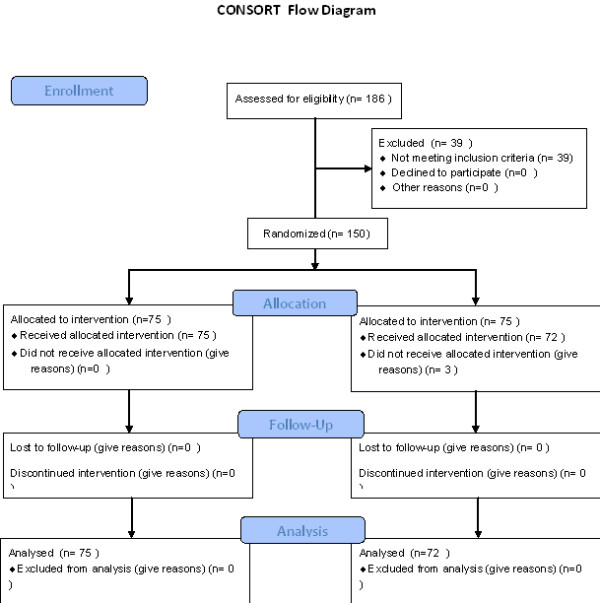
CONSORT flow diagram.

This randomized controlled trial (number IRCT201 107187038N2) was approved by the Ethical Committee of Zahedan University of Medical Sciences and was undertaken in accordance with CONSORT guidelines (Figure 1).

From December 2007 to June 2008, 147 students residing in the dormitory of Zahedan University in south-East Iran were enrolled in the study.

Inclusion criteria were age 18 to 30 years, and dysmenorrhea in young women within the first 2 years of the onset of menstruation and all had regular menstrual cycles and good general health, moderate and severe cramps.

Exclusion criteria: not history of endometriosis [because rate of endometriosis is high [19] and an history of endometriosis can justify pain also in patients who were surgically treated, both with eventual Oral estroprogestins after primary surgery and without adjuvant oral therapy post surgery [20], or other any underlying illness or cause. The students were randomly probably sampling assigned into ibuprofen and heat patch groups.

The students were asked two questions (based on suggestions from The Association of Women's Health, Obstetric and Neonatal Nurses) to screen for dysmenorrhea: 1) Have you ever experienced pelvic pain or cramps during or around menstrual bleeding? and 2) Was the pain bearable for you?

Of the 186 women initially enrolled in our study, 39 were excluded. Exclusion criteria were: a history of co-morbidities (e.g., cardiovascular, renal, hepatic, or pulmonary disease), coagulopathy, diabetes mellitus, anaemia, gastrointestinal bleeding, immunological disorder, malignancy, psychiatric illness requiring therapeutic intervention, use of contraceptive pills, smoking, history of childbirth, professional athletic activity, lower abdominal scars, body mass index (BMI) >30 kg/m^2^, history of vaginitis, and recent death or stressful event in the immediate family.

Abdominal circumference and BMI were measured before the intervention. In the heat patch group (n = 72), a 7 × 12 cm patch (Mahdad Afruz Co., Iran) was supplied to the participants to place in their underwear when menstrual bleeding started. The students were trained in the use of the patch. In the ibuprofen group (n = 75), ibuprofen was prescribed (400 mg, orally every 8 hours, as required).

The students were asked to complete two questionnaires. One questionnaire collected demographic data, and the other was a shortened revision of the McGill Pain Questionnaire (SF-MPQ). The students were given instruction on completing the pain questionnaire. The severity of pain was assessed using 15 descriptive phrases. Eleven phrases described sensory aspects of pain giving a score from 0 to 33 points, and four phrases described emotional aspects of pain giving a score from 0 to 12 points. Current and overall pain severity scores were indicated using the visual analog scale (0–100 and 0–5, respectively). Higher numbers indicated more severe pain. Pain was measured at 2, 4, 8, 12, and 24 hours after the onset of menstruation.

The McGill questionnaire is a reliable tool, with proven validity and reliability, which has been recommended for clinical investigations in the Iranian population [21]. This questionnaire has been also previously been used for the assessment of pain in dysmenorrhea [22]. The validity of McGill questionnaire was evaluated in 1975 by Melzack [23], and the validity of the SF-MPQ has been evaluated for obstetric, postoperative, physical therapy, and dentistry patients. The shortened version has been compared with the standard McGill questionnaire and a significant correlation between the two has been demonstrated [24].

SPSS software version 13 was used for statistical analysis. The student's *t* test was used to compare measurements between the groups.

## Results

### Demographic characteristic

The mean age of the students in the heat Patch and ibuprofen groups was 22.66 and 20.92 years, respectively (P > 0.05). There was also no significant difference between the groups regarding the marital and socio-economical status, mean body mass index, and abdominal circumference75 (93.1%) of the students in the heat patch group and72 (90.7%) of the students in the ibuprofen group were married (Table 1).

**Table 1:** Demographic characteristic two groups

**BMI(weight/high**^**2**^**)**		**Abdomen circumference(cm)**		**Age(year)**		
**Mean**	**SD**	**Mean**	**SD**	**Mean**	**SD**	
2.59	20.96	8.66	76.88	2.91	22.66	**Heat pach**
3.26	20.61	9.71	76.96	2.04	20.92	**Ibuprofen**
P > 0.05		P > 0.05		P > 0.05		*T*-TEST

### Pain domains

The maximum and minimum pain severities were observed at 2 and 24 hours after menstruation in both groups. The severity of sensual pain at 8, 12, and 24 hours after menstruation was non-significantly less in the heat Patch group. The severity of emotional pain impact and final assessment mean of the total pain rating at 4, 8, 12, and 24 hours were not significantly different (Tables 2, Additional file 1: Table S3).

**Table 2:** Comparison score (mean ± SD) pain domains McGill primary dysmenorrheal in two groups

**Pain Domain**	**Score**	**Ibuprofen score**		**Heat patch score**		**p/value**
		**SD**	**Mean**	**SD**	**Mean**	
Severity sensual pain	(0–33)	6.84	5.55	6.81	5.51	P > 0.05
Severity emotional pain	(0–12)	2.94	3.13	2.60	2.63	P > 0.05
Severity Current pain	(0–100)	32.91	26.97	36.41	26.54	P > 0.05
Total pain	(0–5)	2.72	3.57	1.93	1.63	P > 0.05

## Discussion

In this study, the heat wrap group had milder pain during the first 24 hours of menstruation compared with the ibuprofen group, but this difference was not statistically significant.

Increased attention has recently been paid to non-pharmacological treatments for dysmenorrhea [25,26]. However, only a few studies have addressed this issue. Akin and colleagues showed that local heat was as effective as ibuprofen for the treatment of dysmenorrhea [16,27]. However, local heat was not shown to be effective in other reports [12]. A new thermogenic device has also been evaluated which provides a constant temperature of 40 °C for 8 hours through the oxidation of iron chips, which does not interfere with daily activities and is effective, safe, and reasonably priced [13].

In our study, pain was measured using the SF-MPQ. This questionnaire has three main sections and contains a number of phrases to assess the individual’s pain perception (sensual and emotional). Cultural differences, socioeconomic factors, the availability of support, and the individual’s personality play an important role in pain perception. The McGill questionnaire takes these into consideration. Bajaj and colleagues also used this tool to compare the effects of local heat on the pain threshold of women with and without dysmenorrhea and found that dysmenorrhea pain could be soothed by applying local heat, and that pressure on the abdomen and back increased the pain threshold [22]. In our study, we applied heat to only one point. We found that the total pain rating was less in the local heat group, although there were no significant differences between groups.

There were noticeable differences in the emotional aspects of pain during the first 12 hours after the onset of menstruation, but these were not statistically significant. In this the current pain score appears was low in Ibuprofen group than in heat patch group at 8 h, because the maximum effectiveness pad is 8 hours. But This difference was not significant (t = 1.18, p = 0.24). With a larger sample size, this difference may have become significant. Akin and colleagues compared the effectiveness of oral ibuprofen or placebo with and without local heat, and observed that ibuprofen with and without local heat was more effective than placebo. Local heat (non-thermal wrap) was also found to be more effective than placebo. The analgesic effect was significantly faster in the ibuprofen plus local heat group than in the other groups [28].

There are some notable differences between our study and that by Akin and colleagues. We used an 84 cm^2^ patch and the multidimensional McGill pain score tool, and Akin used a 180 cm^2^ wrap and a one-dimensional visual analog pain scale, but these differences should not affect the results. Both studies applied local heat to the same area of the abdomen.

Some of our results are consistent with those of Bajaj and colleagues, who reported that a towel containing an iron chip was more effective in reducing back pain than ibuprofen and acetaminophen on the first, third, and fourth days of menstruation, which was probably due to the large surface area of application and to the area where the heat was applied. This effect could be due to pressure-sensitive points on the back. In comparison with acetaminophen, these towels have been shown to be more effective in the treatment of dysmenorrhea at 3, 4, 5, and 6 hours after the onset of menstruation [27]. Some studies have shown that transcutaneous electrical nerve simulation, which works by the same mechanism as local heat, can be effective for the treatment of dysmenorrhea. This method places stimulatory electrodes over the lumbar and sacral areas, together with local heat. Further investigation of this method is needed [29]. Heat wraps have also been compared with acetaminophen, and were found to be significantly more effective [27].

Although pharmacotherapy can be used to treat dysmenorrhea, it is not effective in 20–25% of patients [6]. Using NSAID drugs as first-line treatment may be limited because of adverse reactions such as gastrointestinal side effects, which mandates the selection of alternative therapeutic modalities.

A retrospective study of the complications of dysmenorrhea showed that 98% of teenagers had used one or more methods to control the pain [30]. In another study, all participants had used local heat to treat the pain [31]. The physiological effects of thermal therapy act via nervous, vascular, and biophysical pathways. Analgesia via the nervous pathway can be explained by Melzack's gate-control theory of pain. [25,26]. This theory provides a logical explanation for the use of pain control measures such as local heat, cold, pressure, massage, and electrical stimulation [24]. These findings are compatible with the findings of Akin and Nadler [16,27,28].

Heat induced vascular reactions will increase the blood flow to an area, resulting in the dilution of intravascular prostaglandins, bradykinin, and histamine. These molecules are among the most potent pain inducing molecules. Increased blood flow also improves tissue oxygenation [32]. Local heat applied to the upper abdomen increases gastrointestinal motility and has a relaxing effect on the uterus. Previous studies have provided explanations for this mechanism [16]. NSIADs are the standard medications used to treat dysmenorrhea. Local heat is as effective as NSIADs [16], but users should be aware of potential side effects of NSAIDs.

Few studies have addressed the use of ibuprofen for dysmenorrhea in Iran [33-35]. Our study is the first which compares the effectiveness of this medication with local heat. There are some studies from other countries which evaluate NSAIDs for the treatment of dysmenorrhea, but according to a review study, there is little or no evidence regarding the safety and adverse effects of these drugs [36]. We found that this apparently insignificant result could open a new scenario for women who can’t be treated by FANS (e.g. allergy, haemorrhagic diathesis, gastric ulcera).

Our study has some limitations, including the effects of variations in the thickness of lower abdominal adipose tissue which acts as a thermal insulator [37] However, abdominal circumference and BMI were similar between groups. Blinded, double blinded, or triple blinded treatments were not possible in our study since the therapeutic measures used were obvious. The pain threshold varied among subjects, because people experience pain differently in different emotional states, which was beyond the control of the investigators. We excluded women with a history of psychiatric or personality disorders and those who were grieving to address this issue.

## Conclusions

The main purpose of any treatment is to provide the most effective and less harmful therapeutic methods for the patients. We introduced a new tool for pain control which can possibly replace ibuprofen in the treatment of primary dysmenorrhea. We showed that probably heat Patch containing iron chip has comparable analgesic effects to ibuprofen, Because there wasn't significant. And as a complementary and non-drug methods may be proposed.

## Competing interests

The authors declare that they no competing interests.

## Authors' contributions

SHDNR. conceived of the study, and participated in its design and coordination and helped to draft the manuscript FKS participated in the sequence alignment and drafted the manuscript AN participated in the design of the study and performed the statistical analysis LS participated in the Criticality revised the manuscripts AS and SKH and SHSH and TS were involved data collection collection and.Administration of satisfaction questionnaire and provided and the primary draft manuscript and other authors read and approved the final manuscript .All authors read and approved the final manuscript.

## Pre-publication history

The pre-publication history for this paper can be accessed here:

http://www.biomedcentral.com/1472-6874/12/25/prepub

## Supplementary Material

Additional file 1 Table S3: Compare mean score pain in 2,4,8, 12 and 24hr after treatment two goups (Heat iron pach-Ibuprofen). Click here for file
