# Supplementary material for: Comparing the analgesic effect of heat patch containing iron chip and ibuprofen for primary dysmenorrhea: a randomized controlled trial
Source: BMC Womens Health. 2012 Aug 22;12:25. doi: 10.1186/1472-6874-12-25 (PMC3492023; doi:10.1186/1472-6874-12-25)
Supplement: Additional file 1 — Table S3: Compare mean score pain in 2,4,8, 12 and 24hr after treatment two goups (Heat iron pach-Ibuprofen). [file 1472-6874-12-25-S1.doc]

# Tables

|  | **Age(year)** | | **Abdomen circumference(cm)** | | **BMI(weight/high^2^)** | |
| --- | --- | --- | --- | --- | --- | --- |
|  | Mean | SD | Mean | SD | Mean | SD |
| **Heat pach** | 22.66 | 2.91 | 76.88 | 8.66 | 20.96 | 2.59 |
| **Ibuprofen** | 20.92 | 2.04 | 76.96 | 9.71 | 20.61 | 3.26 |
| T-TEST | P>0.05 | | P>0.05 | | P>0.05 | |

## Table 1 - Demographic characteristic two groups

|  | | **Heat pach** | | **ibuprofen** | |  |
| --- | --- | --- | --- | --- | --- | --- |
|  |  | score | | score | |  |
|  |  | Mean | SD | Mean | SD |  |
| Severity sensual pain | (0-33) | 6.81 | 5.51 | 6.84 | 5.55 | P>0.05 |
| Severity emotional pain | (0-12) | 2.60 | 2.63 | 2.94 | 3.13 | P>0.05 |
| Severity present pain | (0-100) | 36.41 | 26.54 | 32.91 | 26.97 | P>0.05 |
| Total pain | (0-5) | 1.93 | 1.63 | 2.72 | 3.57 | P>0.05 |

**Table 2 -** **Comparison score(mean±SD)Pain domains Mcgill primary**

**dysmenorrheal in two groups**
